# Injectable photocrosslinking spherical hydrogel-encapsulated targeting peptide-modified engineered exosomes for osteoarthritis therapy

**DOI:** 10.1186/s12951-023-02050-7

**Published:** 2023-08-21

**Authors:** Junlai Wan, Zhiyi He, Renpeng Peng, Xiaopei Wu, Ziqing Zhu, Jiarui Cui, Xiaoxia Hao, Anmin Chen, Jiaming Zhang, Peng Cheng

**Affiliations:** 1 Department of Orthopedics, Tongji Hospital, Tongji Medical College, Huazhong University of Science and Technology, 1095#, Jie-Fang Avenue, Qiaokou District, Wuhan, 430030 Hubei China; 2 State Key Laboratory of Advanced Technology for Materials Synthesis and Processing, Wuhan University of Technology, Wuhan, 430070 China; 3 Longhua Hospital, Shanghai University of Traditional Chinese Medicine, Shanghai, 200032 China; 4 Department of Rehabilitation, Tongji Hospital, Tongji Medical College, Huazhong University of Science and Technology, Wuhan, 430030 China; 5 Division of Spine Surgery, Department of Orthopedic Surgery, Nanjing Drum Tower Hospital, Affiliated Hospital of Medical School, Nanjing University, Nanjing, 210008 China

**Keywords:** Osteoarthritis, Exosome, Surface modification, Hydrogel, Nanomedicine

## Abstract

Osteoarthritis (OA) is a common degenerative joint disease urgently needing effective treatments. Bone marrow mesenchymal stromal cell-derived exosomes (Exo) are considered good drug carriers whereas they have limitations such as fast clearance and low retention. This study aimed to overcome the limitations of Exo in drug delivery using multiple strategies. Novel photocrosslinking spherical gelatin methacryloyl hydrogel (GelMA)-encapsulated cartilage affinity WYRGRL (W) peptide-modified engineered Exo were developed for OA treatment and the performance of the engineered Exo (W-Exo@GelMA) loaded with a small inhibitor LRRK2-IN-1 (W-Exo-L@GelMA) was investigated in vitro and in vivo. The W-Exo-L@GelMA showed an effective targeting effect on chondrocytes and a pronounced action on suppressing catabolism and promoting anabolism in vitro. Moreover, W-Exo-L@GelMA remarkably inhibited OA-related inflammation and immune gene expression, rescuing the IL-1β-induced transcriptomic responses. With enhanced retention in the joint, W-Exo-L@GelMA demonstrated superior anti-OA activity and cartilage repair ability in the OA murine model. The therapeutic effect was validated in the cultured human OA cartilage. In conclusion, photocrosslinking spherical hydrogel-encapsulated targeting peptide-modified engineered Exo exhibit notable potential in OA therapy. Engineering Exo by a series of strategies enhanced the targeting ability and retention and cartilage-targeting and Exo-mediated drug delivery may offer a novel strategy for OA treatment.

*Clinical trial registration*: Not applciable.

**Graphical Abstract:**

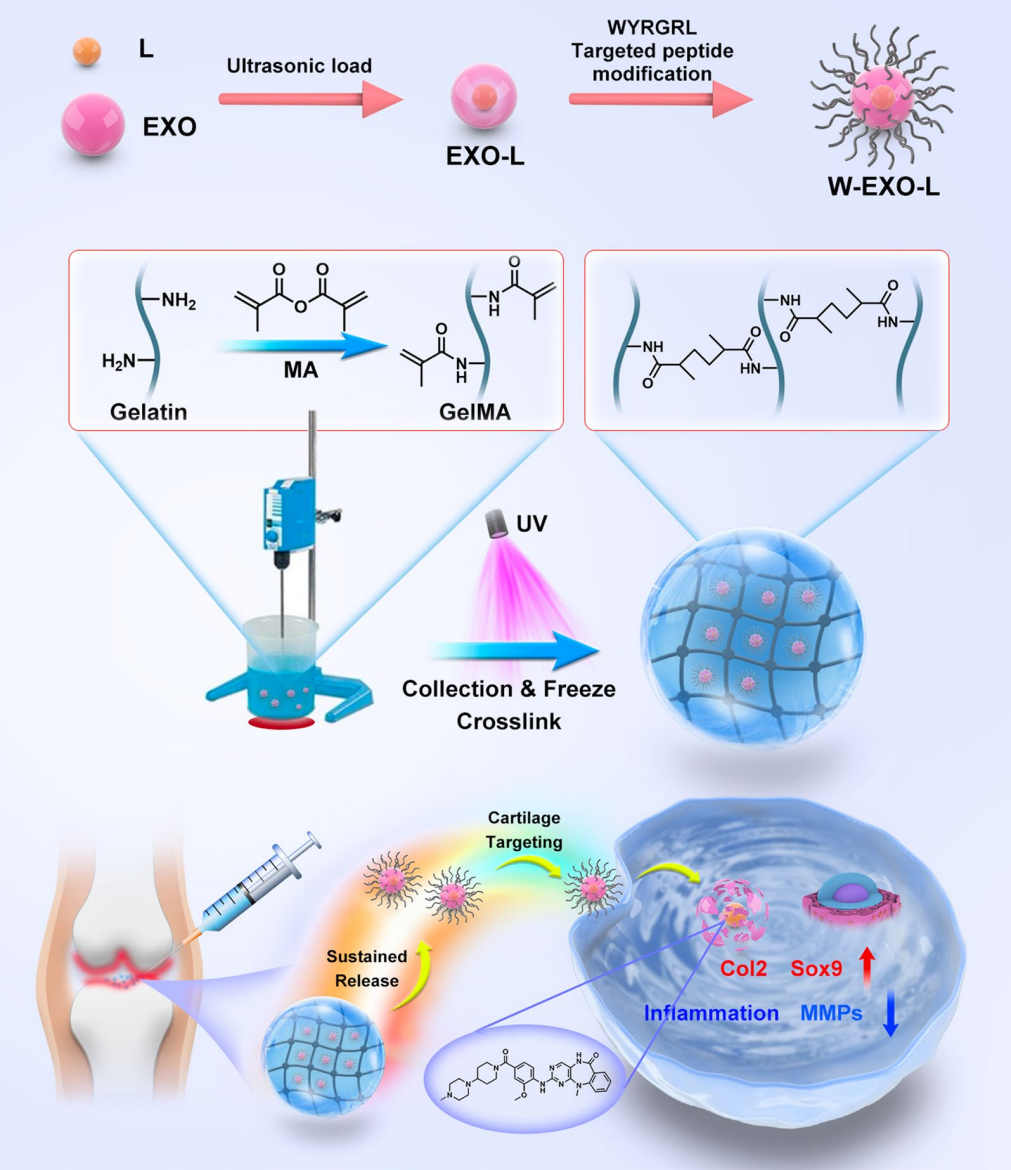

**Supplementary Information:**

The online version contains supplementary material available at 10.1186/s12951-023-02050-7.

## Introduction

Articular cartilage is a thin layer of transparent connective tissue that covers the surface of the joint to support its movement [1]. Since chondrocytes are highly terminal differentiated cells, injured or aged chondrocytes have a very limited potential for self-repair [2]. The repair of articular cartilage defects caused by post-traumatic and degenerative osteoarthritis (OA) has become a worldwide challenge. As the cornerstone of OA management, topical or oral nonsteroidal anti-inflammatory drugs (NSAIDs) can partially relieve the clinical symptoms, whereas NSAIDs cause side effects and cannot impede OA progress [3–5]. Emerging novel therapies, including disease-modifying drugs, have the potential in OA treatment [6, 7], but none of them has been proven to modify disease progression significantly or successfully prevent final joint replacement in the advanced disease stage [8]. Thus, establishing effective treatment strategies that can induce the repair and regeneration of articular cartilage is be of substantial usefulness for OA.

Conventional systemic or intra-articular administrations cause low accumulation and retention of drugs in joints, causing reduced drug efficacy and dose-limiting toxicities [9]. The limitations of the pharmacokinetics of traditional drug delivery systems, such as fast drug clearance, attenuate the actual therapeutic effects on OA cartilage [10]. Recent advances in nanomedicine-based delivery strategies, such as intra-articular injections of engineered drug-loaded micro- and nano-particles, liposomes, and hydrogels, shed light on drug delivery in OA treatment [10–12]. Despite the promising effects on OA treatment, a single strategy still has its limitations, thus achieving a composite and pleiotropic drug delivery system with high efficiency of delivery and targeting is an important mission for OA.

Exosomes, as the vesicles secreted by cells, have become an emerging drug carrier due to their unique advantages in these aspects [13, 14]. Due to their natural membrane structure and small size of about 40–100 nm, exosomes are characterized by many unique characteristics, such as their ability to penetrate natural barriers, high cycling stability, and specific targeting, making them excellent vehicles for drug delivery [15, 16]. Engineered exosomes undergoing surface modifications, such as genetic engineering and chemical modifications, exhibited enhanced targeting ability, thus facilitating local concentrations, low immunogenicity, low toxicity, and high engineerability and improving their potential for a wide range of diseases [17, 18]. More than a drug carrier, exosomes derived from mesenchymal stem cells and other cells have their intrinsic actions in attenuating local inflammation, promoting anabolism, and suppressing catabolism, thus restoring OA cartilage [19, 20]. However, their compelling shortcomings, e.g., rapid cellular and enzymatic clearance in joints, have limited the therapeutic effects and application of exosomes. Recent achievements have demonstrated that the combination of biocompatible and highly adhesive biomaterials, such as natural hydrogels, facilitates the retention of exosomes without fast clearance or leakage from the joint [19, 21, 22]. Moreover, although surface modification of exosomes has been proven to improve the targeting of actions in multiple diseases [23, 24], advanced modified exosome-based drug delivery for OA is rarely found.

LRRK2-IN-1, a small molecular inhibitor, inhibits leucine-rich repeat kinase 2 (LRRK2) which controls mitochondrial homeostasis, microtubule dynamics, inflammation, and autophagy [25–28], exhibiting the potential as a potential disease-modifying drug for OA. However, like most inhibitors, LRRK2-IN-1 has disadvantages, such as local cytotoxicity or potential side effects. To ameliorate these limitations and improve the accurate action on cartilage, a novel exosome-based hydrogel delivery system was developed in this study. In the design, a cartilage affinity peptide was used to modify the surface of LRRK2-IN-1-loaded exosomes to improve the targeting ability. Photocrosslinking spherical hydrogel was used to encapsulate them to avoid rapid clearance and destruction. In this study, we developed a targeted peptide-modified exosome-based composite hydrogel for the sustained release of LRRK2-IN-1, demonstrating an advanced drug delivery and the potential of exosome in OA targeted treatment.

## Materials and methods

### Chemicals and reagents

LRRK2-IN-1 (CAS 1234480-84-2) was purchased from Topscience (T2246; China). Recombinant mouse IL-1β cytokine (#501-RL-010) and human IL-1β cytokine (201-LB-025) were obtained from R&D Systems (USA). The primary antibodies against glyceraldehyde 3-phosphate dehydrogenase (GAPDH; #60004-1-Ig), inducible nitric oxide synthase (iNOS; #22226-1-AP), cyclooxygenase 2 (COX2; #66351-1-Ig), type II collagen (COL2; #28459-1-AP), aggrecan (#13880-1-AP), and matrix metallopeptidase 13 (MMP13; 18165-1-AP) were acquired from Proteintech Group (China) and the primary antibodies against matrix metallopeptidase 3 (MMP3; # BM4074) and SRY-box transcription factor (SOX9; #A00177-2) from Boster Biological Technology (China). Trypsin, collagenase type II, phosphate buffer saline (PBS) buffer solution, and the secondary antibodies for western blot and immunofluorescence analyses were provided by Boster Biological Technology (China). In addition, fetal bovine serum (FBS) and Dulbecco’s modified Eagleʼs medium F12 (DMEM/F12) were provided by Gibco (USA). Safranin O solution was provided by Solarbio (#G1067; China).

### Chondrocyte isolation and culture

Chondrocytes were obtained from the knee joints of sacrificed C57BL/6 mice (5 days old) by sequential enzymatic digestion as previously described [29]. Briefly, the total knee cartilage was extracted and placed in PBS. The synovium and other attachments were stripped thoroughly from the cartilage surface. Then, the separated cartilage was crumbled into miniature patches and immersed in 0.25% trypsin for digestion in a cell incubator for 30 min. After centrifugation, the sediment was transferred and regurgitated with 0.2% collagenase II for 6 h in a hybridization oven at 37 °C. Then, the chondrocyte sediment was isolated from suspension by centrifugation at 1500 r/min for 5 min and cultured in a complete medium containing 10% FBS (Gibco, USA), 1% penicillin/streptomycin, and DMEM/F12 culture medium in a cell incubator. Matured chondrocytes were collected for subsequent in vitro experiments. IL-1β (5 ng/mL) was used for the induction of OA-like chondrocyte phenotypes in vitro [4, 30].

### In Vitro cytotoxicity test

In vitro cytotoxicity of LRRK2-IN-1 was measured using the cell counting kit-8 (CCK8) method. In brief, chondrocytes were cultured in 96-well plates (5000–10,000 cells/well) for 24 h, followed by LRRK2-IN-1 treatment for 24 h. The CCK8 reagent was added to the plates and the absorbance value in 450 nm wavelength was detected using a microplate reader (BioTek, USA).

### Western blot analysis

Protein extraction and western blot analysis were performed as the previous studies described [31, 32]. In brief, the cells mixed with radio-immunoprecipitation assay buffer (RIPA) supplied with phosphatase inhibitors and protease inhibitors in the proportion of 100:1:1 (Boster, China) were scraped off with cell wipers and the cell lysis was collected. The cell lysis was treated using an ultrasonic crusher (Sonicator Q125; Qsonica, USA) and centrifuged at 12,000 rpm, 4 °C for 30 min. After centrifugation, the supernatant was collected and added with loading buffer in the proportion of 4:1, refrigerated for 5 min, and denatured at 95 °C for 5 min. The total protein was then subjected to sodium dodecyl-sulfate polyacrylamide gel electrophoresis (8.0–12.5%) and transferred to a polyvinylidene difluoride membrane. The unbound sites on the membrane were blocked with 5% bovine serum albumin for 1 h. The targeted bands were cut out from the membrane and incubated with the specific primary antibodies at 4 °C overnight, and the membranes were rinsed thrice with tris-buffered saline with 0.1% Tween 20 for 15 min, incubated with specific secondary antibodies for 1 h at room temperature, and washed again thrice with tris-buffered saline with 0.1% Tween 20 for 15 min. Protein blots were developed using a Western ECL Substrate Kit (Thermo Fisher Scientific, USA) and Bio-Rad scanner (BioRad, USA). The intensity of bands was quantified by digital image analysis software (Bio-Rad, USA).

### Immunofluorescence staining

Chondrocytes were seeded at 2 × 10^4^ per dish and cultured on dishes for 24 h. These were then stimulated with IL-1β (5 ng/mL) for 24 h with or without LRRK2-IN-1 (5 µM) for 24 h. Next, the plates were fixed with 4% paraformaldehyde and rinsed with 0.2% Triton X-100 for 15 min. Then, the unbound sites on the plates were blocked with 1% bovine serum albumin for 30 min at normal temperature. Subsequently, they were treated with primary antibodies against aggrecan, iNOS, and MMP13 at 4°C overnight. After washing with PBS twice, the chondrocytes were incubated with Cy3-conjugated goat anti-rabbit secondary antibody for 1 h at room temperature in the dark. Finally, the nuclei of chondrocytes were dyed using 4ʹ,6-diamidino-2-phenylindole (DAPI) for 10 min. Screening and photography were performed using a fluorescence microscope (Nikon, USA).

### Isolation of exosomes

Rat bone marrow mesenchymal stromal cells (BMSCs) were harvested and cultured with DMEM medium containing 10% FBS as previously described [33]. When the cell confluency reached 30–50%, the original medium was discarded and DMEM basic medium without FBS was added after the cells were washed with PBS three times. After 48–72 h, the cell culture supernatant was collected and underwent ultracentrifugation to collect exosomes. The detailed steps are as follows: centrifuge at 300 g for 10 min, take the supernatant, and remove the pellets to remove the cells; centrifuge at 2000 *g* for 10 min, take the supernatant, and discard precipitate to remove dead cells; centrifuge at 10,000 *g* for 30 min to remove the cell fragments in the sediment; centrifuge at 100,000 *g* for 70 min by ultracentrifugation; resuspend pellets at the bottom of the centrifuge tubes with a small amount of PBS. The concentration was quantified with a BCA reagent kit (#23225, Thermo Fisher Scientific, USA).

### Drug loading in exosomes

LRRK2-IN-1 and exosomes were mixed in a 1:1 mass ratio. With the probe set as 0.25 mm and the amplitude set as 20%, the mixed solution was treated sequentially by 3 min ultrasound, 30 s ultrasound, and 2 min pause on ice, and these steps were repeated 6 times. After the recovery of the exosome membrane at 37 ℃ for 1 h, the mixed solution of exosomes and LRRK2-IN-1 was centrifuged at 100,000 *g* for 70 min to collect LRRK2-IN-1 loaded exosomes (Exo-L). To measure drug concentration, Exo-L were destroyed using an ultrasonic cleaning machine for 30 min, and centrifuged for 20 min at 4 ℃, 15,000 *g*. After diluting the supernatant with deionized water, the drug content in the exosomes was determined by liquid chromatography. The 20 µL supernatant of the test sample was injected into the HPLC column to detect the elution of the drug. The drug encapsulation efficiency was calculated according to the following formula (Additional file 1: Table S1): The drug encapsulation efficiency = W_b_/W_a _× 100%. Note: W_a_ is the mass of the drug loaded, and W_b_ is the initial system mass of the drug. The drug loading rate was calculated according to the following formula (Additional file 1: Table S2): Drug loading rate = W_1_/W_0_ × 100%. Note: W_1_ is the mass of the drug loaded, and W_0_ is the total mass of the drug loaded exosomes.

### Target peptide-modification of exosomes

Drug-loaded exosomes were dissolved in 15 mL HEPES buffer solution (#15630080, Gibco, USA). 2 µL methyl acrylic anhydride (276685, Sigma, Germany) was added into the mixed solution which was then stirred for 24 h. The mixed solution was filtered by a 0.22 μm filter to remove the precipitated acrylic acid. Methacrylic anhydride was acylated with the amino groups on the surface of exosomes to form olefin double bond modified exosomes. The exosomes were collected again by ultracentrifugation, dissolved in 15 mL HEPES buffer, added with 1 mg peptide WYRGRL (Additional file 1: Fig. S1), mixed well, placed at 0 ℃, and stirred for 24 h. Alkene double bond modified exosomes underwent acylation reactions with amino groups in targeted peptides, achieving the binding of peptides to exosomes. The peptide segments were combined with the exosome membrane. The superfluous unconnected peptide segments were removed by ultrafiltration tube, and the peptide-modified exosomes (W-Exo-L) were collected by ultracentrifugation. The formulation of resulted peptide-modified exosomes was resuspended in phosphate buffer for quantification and freezing.

### Characterization of target peptide-modified exosomes

The total protein of BMSCs and exosomes was extracted using RIPA lysis (#89900, Thermo Fisher Scientific, USA). In brief, BMSCs and exosomes were added with RIPA lysate containing PMSF (#36978, Thermo Fisher Scientific, USA), incubated on ice for 30 min at 4 ℃, and centrifuged at 8000 *g* for 10 min. The supernatant was taken and the total protein concentration was detected with the BCA kit. After 20 µg protein was dissolved in the loading buffer and incubated in a 100 ℃ metal bath for 5 min, the protein samples were analyzed by western blot analysis to detect the expression of the exosome marker proteins including TSG101 (#28283-1-AP, Proteintech, China), CD81 (#27855-1-AP, Proteintech, China), and CD9 (#20597-1-AP, Proteintech, China). The isolated and purified exosomes were quickly taken out, dropped onto the copper mesh, and fixed in 5% glutaraldehyde with protection from light. The exosomes were negatively stained with 2% phosphotungstic acid in 0.1 M phosphate buffer at 4 ℃, and analyzed by transmission electron microscope (TEM, HT-7700, Hitachi, Japan) at 100 KV. 10 µg exosomes were dissolved in 1 mL PBS, swirled and shaken, and then mixed well. Then, the particle size distribution and surface charge of exosomes were directly observed and measured with a Nanoparticle Tracking Analyzer.

### Photocrosslinking spherical hydrogel encapsulation of targeting peptide-modified exosomes

The methacrylate gelatin (GelMA) (#900629, Sigma, Germany) solution with a concentration of 20% was prepared, and 300 µg/mL exosomes modified with targeted peptides were added to 1 g GelMA and mixed uniformly at 40 ℃. The liquid paraffin span-80 (10:1) (8.40123, Sigma, Germany) was added to the round bottom flask and stirred at 350 r/min for 10 min to obtain a well-mixed emulsion, which was used as the oil phase for standby. The mixed solution of GelMA and exosomes were slowly added to the oil phase which was then stirred and emulsified at a certain speed for 10 min. Then the mixed solution was stirred in a ~ 4 ℃ ice water bath and added with 200 µL LAP photoinitiator (#900889, Sigma, Germany) for photocrosslinking. After crosslinking, the mixed solution was added with isopropanol to generate flocculation, which was further filtered, washed by isopropanol for 3 times to remove the un-encapsulated exosomes, and dried to obtain the exosomes encapsulated with GelMA microspheres (W-Exo-L@GelMA).

### Encapsulation efficiency and loading capacity of exosomes into GelMA

The exosomes loaded GelMA prepared using the above method were destroyed using an ultrasonic cleaning machine for 30 min, and centrifuged for 20 min at 4 ℃, 15,000 *g*. After diluting the supernatant with deionized water, the protein content was determined through the BCA protein quantification kit (Thermo Fisher Scientific). Briefly, 25 µL standard and diluted test samples were added to the 96 well plate and 200 µL of BCA working solution was added to a 96 well plate and incubated at 37 ℃ for 30 min. The sample was measured by a multifunctional enzyme-linked immunosorbent assay under the absorbance conditions of A562. According to the measured values of the standard sample, the standard curve is drawn and the protein concentration of the sample to be tested is calculated using the standard curve. The exosomes encapsulation efficiency was calculated according to the following formula (Additional file 1: Table S3): The exosome encapsulation efficiency = W_b_/W_a _× 100%. Note: W_a_ is the mass of the exosome loaded, and Wb is the initial system mass of the exosome. The exosome loading rate was calculated according to the following formula (Additional file 1: Table S4): Drug loading rate = W_1_/W_0_ × 100%. Note: W_1_ is the mass of the exosome loaded, and W_0_ is the total mass of the exosome-loaded GelMA.

### Characterization of spherical hydrogel

The spherical hydrogel was observed under an inverted fluorescent microscope (Olympus IX71, Olympus, Japan). The chemical composition was analyzed by Fourier-transform infrared spectroscopy (FT-IR) (Nicolet 6700, Thermo Fisher Scientific, USA) in the 400–4000 cm^−1^ range. The morphology of the hydrogel microsphere was observed by scanning electron microscopy (SEM) (Zeiss, Gemini300, Germany). The hydrogel microsphere was added to the PBS solution and stirred at 37 ℃. 100 µL test samples were taken out from the dialysis bag at each time point, followed by 100 µL PBS solution supplement. The test samples were heated at 75 ℃, vortexed, and centrifuged at 13,000 rpm for 10 min. After filtration, 20 µL supernatant of the test sample was injected into the HPLC column to detect the elution of the drug. The release of LRRK2-IN-1 was expressed as a percentage of the total amount of LRRK2-IN-1. In vitro cytotoxicity of W-Exo-L@GelMA treatment for 48 h was measured using the CCK8 method.

###  Endocytosis of Dil-labeled W-Exo-L@GelMA by chondrocytes

The extracted Exo were diluted with PBS, blended in Dil staining solution (500:1, D3911, Invitrogen, USA), and incubated at 37 ℃ away from light for 15 min. Then, the samples were centrifuged at 100,000 *g* for 60 min to discard the supernatant and cleaned with PBS 3 times to remove the redundant staining solution. The labeled Exo was used to prepare W-Exo-L@GelMA and then were co-cultured with chondrocytes for 48 h. The chondrocytes were immersed 3 times in PBS, and fixed by 4% paraformaldehyde (Gibco, USA) for 10 min. After washing 3 times with PBS, the fixed cells are immersed in phalloidin liquid (A12379, Alexa Fluo 488, Invitrogen, USA) for 15 min. Subsequently, 4ʹ, 6-diamidino-2-phenylindole (DAPI) (D1306, Invitrogen, USA) was added into the fixed cells. Eventually, images were observed and collected with a fluorescence microscope (Olympus Optical Co., Ltd, Tokyo, Japan).

### RNA sequencing and bioinformatic analysis

Chondrocytes were treated with IL-1β (5 ng/mL) and with or without W-Exo-L@GelMA loaded with 5 µM LRRK2-IN-1 for 48 h. Total RNA was isolated using Trizol (Invitrogen, USA). In the design of the RNA sequencing (RNA-seq) study, two biological replicates were included in each group. After a quality check, 500 ng RNA was used to construct cDNA libraries, which were subsequently sequenced using the Illumina HiSeq 4000 platform following standard protocols [34]. After trimming adapters and removing reads with low-quality bases (Q ≤ 20) using the fastp tool (v0.18.0) [35], paired-end clean reads were obtained and mapped to the reference genome of Mus musculus GENCODE Release M31 (GRCm39) using HISAT2 (v2. 2.4) [36] with default parameters. The mapped reads were assembled by using StringTie (v1.3.1) [37]. The FPKM (fragment per kilobase of transcript per million mapped reads) value was calculated to quantify the expression abundance of genes using RSEM software (v 1.2.31) [38]. Principal component analysis (PCA) was employed to determine the overall difference in transcriptome between each group. The differential expressed genes (DEGs) were identified by the criteria of FDR < 0.05 using the R package *DESeq2* (v1.38.2) [39]. To identify the robustly-regulated genes, expression variance was defined by the absolute value of the difference in normalized gene expression (FPKM) between the two groups. To identify the rescued effects of LRRK2-IN-1 in gene expression, the up- or down-regulated genes were sorted in descending order of expression variance values in the comparisons between the IL-1β and VEH groups or between the W-Exo-L@GelMA/L-1β and IL-1β groups and normalized rescue scores were defined by the estimates using the R package *RobustRankAggreg* (v1.1) [40] to define the polarized genes in both comparisons. Kyoto Encyclopedia of Genes and Genomes (KEGG) enrichment analyses were performed using the R package *clusterProfiler* (v3.11) [41]. Data visualizations were conducted by R package *pheatmap* (v1.0.12) and *ggplot2* (v 3.4.0) [42, 43].

### RT-qPCR

Trizol reagent (Takara, Japan) was used to extract RNA from cultured cells and then 1 µg total RNA was converted to cDNA using the HiScript II 1st Strand cDNA Synthesis Kit (Vazyme, China). RT-qPCR was performed using primers and templates mixed with the Maxima SYBR Green qPCR Master Mix (Thermo Fisher Scientific, USA) via a CFX Connect RT-qPCR detection system (Bio-Rad, USA). The mRNA levels of genes were normalized to *Gapdh* mRNA in the same sample, and the relative expression of the genes of interest was determined using the formula of Livak and Schmittgen [44]. The primers used for RT-qPCR in this study are listed in Table 1.

**Table 1:** The primers used for RT-qPCR in this study

Gene Symbol	Forward	Reverse
*Saa3*	AGAGAGGCTGTTCAGAAGTTCA	AGCAGGTCGGAAGTGGTTG
*Mmp3*	TCTGGGCTATACGAGGGCAC	ACCCTTGAGTCAACACCTGGA
*Cxcl5*	GTTCCATCTCGCCATTCATGC	GCGGCTATGACTGAGGAAGG
*Hp*	GCTATGTGGAGCACTTGGTTC	CACCCATTGCTTCTCGTCGTT
*Cxcl1*	ACTGCACCCAAACCGAAGTC	TGGGGACACCTTTTAGCATCTT
*Mmp13*	TGTTTGCAGAGCACTACTTGAA	CAGTCACCTCTAAGCCAAAGAAA
*Col3a1*	CTGTAACATGGAAACTGGGGAAA	CCATAGCTGAACTGAAAACCACC
*Dcn*	ACCTCTCGTGAAGTTGGAAAGG	CCCAGAGTTTTTCAGTGGGTTG
*Cxcl12*	TGCATCAGTGACGGTAAACCA	CACAGTTTGGAGTGTTGAGGAT
*Cfb*	TACCCCGTGCAGACTCGAA	GTGGGCAGCGTATTGCTCT
*Vnn1*	GATTCCCAGGGTAAACTGGTTG	CGAAAGTCACAAACTCAGGCT
*Ccl5*	GCTGCTTTGCCTACCTCTCC	TCGAGTGACAAACACGACTGC

### Animal study

The animal study was conducted following the instructions and approval of the Ethics and Animal Research Committee of Huazhong University of Science and Technology. 120 C57BL/6J mice (8-week-old, male) were supplied by the Experimental Animal Centre of Tongji Hospital and fed in the SPF animal laboratory. The mice were randomly assigned into six groups (Sham, DMM, DMM + L@GelMA, DMM + Exo@GelMA, DMM + Exo-L@GelMA, and DMM + W-Exo-L@GelMA). After inhalation-induced anesthesia with isoflurane, the OA model was established by the surgical destabilization of the medial meniscus (DMM) and the Sham group underwent an opening of the joint cavity and excision of only the anterior fat pad. The concentration of LRRK2-in-1 interfering with mouse chondrocytes is 5 µM in vitro, the drug concentration usually injected into mouse joints is 10–30 times than that of in vitro cell experiments, i.e. 50–150 µM, equivalent to 28.535–85.605 µg/mL. According to the sustained release curve of the drug, the cumulative release of the drug (800 µg/mL) within 15 days is 84% (672 µg/mL), an average of 45 µg/mL per day, which is always at the effective concentration. When the total amount of drug (10 µL×800 µg/mL = 8 µg) were determine based on the encapsulation efficiency of exosomes into GelMA (Additional file 1:  Table S4), we could calculate that the total amount of microspheres coated with drug exosomes (47.84 µg). Therefore, on the third day after the surgery, 10 µL L@GelMA (800 µg/mL LRRK2-IN-1), Exo@GelMA, Exo-L@GelMA (800 µg/mL LRRK2-IN-1), or W-Exo-L@GelMA (800 µg/mL LRRK2-IN-1) were injected into the joint cavity, respectively, every two weeks for 8 weeks. Simultaneously, mice in the Sham and DMM groups received 10 µL saline solution. After completing the intra-articular injection course, all the mice were sacrificed and the right knee joints were collected for further evaluation.

### Micro-computed tomography scanning

Knee joints were fixed with 4% paraformaldehyde before scanning. Scanning was conducted using Scanco vivaCT 40 micro-computed tomography (µCT) instrument (Scanco Medical, Switzerland). Parameters were set for calcified tissue visualization as below: 100 kV of source voltage, 98 µA of current, 10 μm voxel size, and 300 ms integration time. Subchondral bone parameters of the tibias including trabecular bone volume fraction (BV/TV), thickness (Tb. Th), spacing (Tb. Sp), and number (Tb. N) were measured as previously described [45].

### Histological staining and immunohistochemistry analysis

After the fixation in 4% paraformaldehyde for 24 h, the knee joints were decalcified in 10% ethylenediaminetetraacetic acid for 30 days, embedded in paraffin, and sliced into 5 μm-thick sections for further tissue staining and immunohistological analysis. Safranin O/fast green staining was used for the histological analysis of cartilage lesions. The degree of cartilage damage was assessed according to the Osteoarthritis Research Society International (OARSI) scoring guidelines [45]. Moreover, after the sections were subjected to deparaffinization and blocked with 5% bovine serum albumin for 1 h, they were incubated with the primary antibodies against anabolic factor aggrecan (13880-1-AP, 1:200) and catabolic factor MMP13 (18165-1-AP, 1:200) at 4 °C overnight. The sections were incubated with secondary antibodies, developed, and observed under a microscope. The human OA cartilage samples were subjected to similar protocols of immunohistochemistry analyses to analyze the expression of aggrecan and MMP13.

### Human cartilage treatment

Human cartilage harvest and treatment were approved by the Human Ethics Committee of Tongji Medical College, Huazhong University of Science and Technology (Wuhan, China) (approval number: TJ-IRB20220947). Three OA patients (mean age, 69 years; range, 61–75 years; male: 2, female: 1) without other systemic diseases were included, who accepted total knee arthroplasty (TKA) at the Department of Orthopedics, Tongji Hospital, Tongji Medical College, Huazhong University of Science and Technology in 2022. Written informed consent of the human tissue harvest was acquired from each patient. Briefly, the medial femoral condyles were isolated during surgery and transformed on ice to the orthopedics laboratory.

The soft tissue clearance in the cartilage surface was completely removed after being washed three times using cold PBS. The cartilage explants sized in 5 × 5 × 5 mm^3^ were excised using a surgical saw and cultured in an incubator containing 5% CO_2_ at 37 °C with DMEM/F12 medium supplemented with 10% FBS, 1% penicillin/streptomycin, and human IL-1β (10 ng/mL). The cartilage explants from the same patient were treated with or without W-Exo-L@GelMA loaded with 5 µM LRRK2-IN-1 for 72 h, fixed in 4% paraformaldehyde at 4 °C for 24 h, and decalcified in 10% EDTA for 30 days for immunohistochemistry analysis.

### Statistical analysis

The experimental data were analyzed using GraphPad Prism v.8.4.0 software (GraphPad, USA). The results are shown as the mean ± SD. The differences between any two groups were determined by the student’s t-test. One-way analysis of variance (ANOVA) followed by Tukey’s test was used to compare differences among two or more groups. A P < 0.05 was recognized as significant. All data were repeated independently at least three times.

## Results

### LRRK2-IN-1 suppresses the IL-1β-induced inflammation and catabolism and induces anabolism without inhibiting chondrocyte viability

**Fig. 1:**
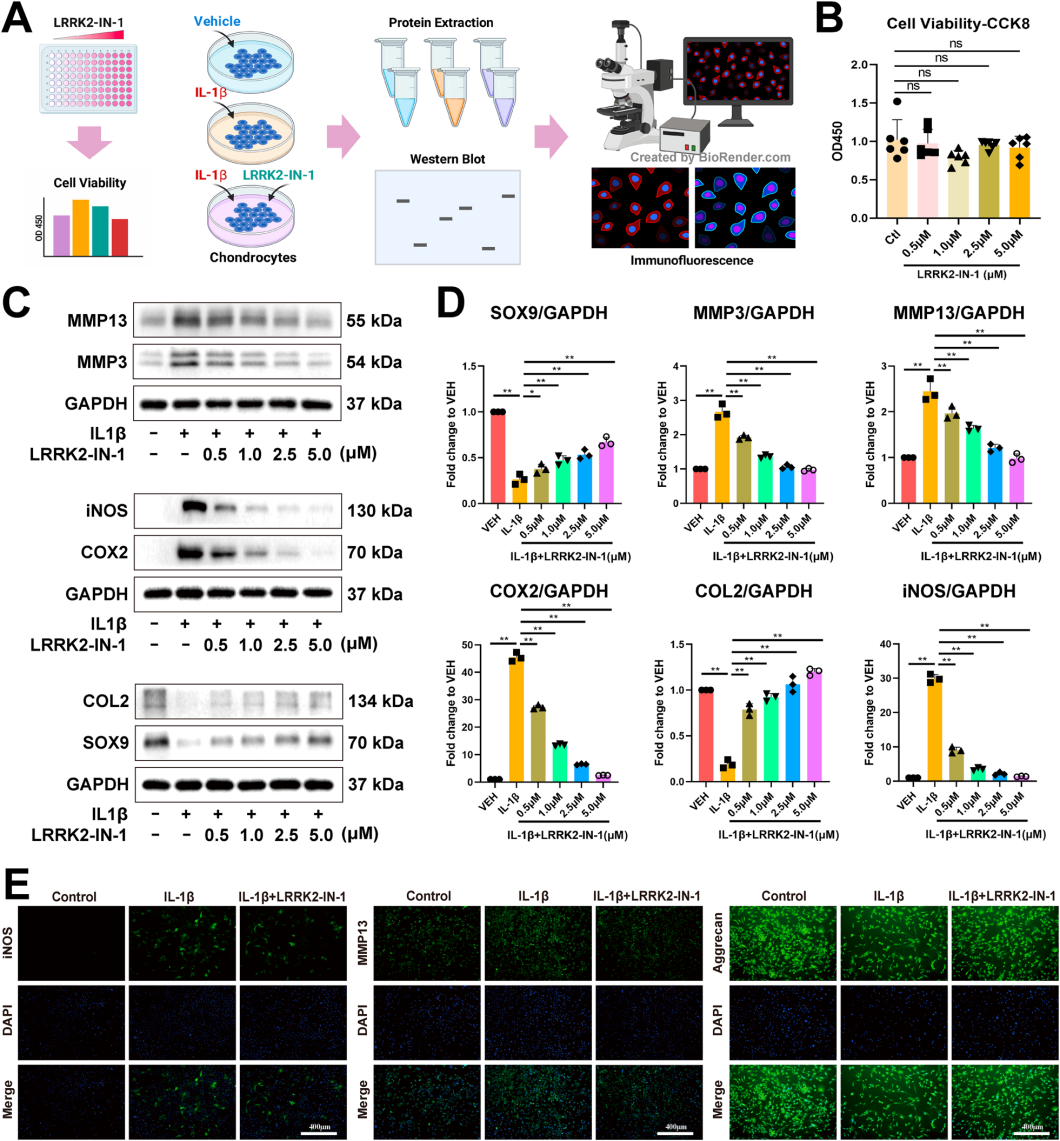
LRRK2-IN-1 suppresses the IL-1β-induced inflammation and catabolism and induces anabolism without causing the inhibition of chondrocyte viability. **A** Schematic diagram of cell treatment and experimental procedures. **B** Cell viability assessed by CCK8 assay. No obvious inhibition of chondrocyte proliferation was observed when treated with 0.5, 1.0, 2.5, and 5.0 µM LRRK2-IN-1 for 24 h. Data represent mean ± SD; N = 6/group; one-way ANOVA; ns, not significant. **C** Western blot analyses of the protein levels of anabolic, catabolic, inflammatory factors in the IL-1β-induced chondrocytes treated with 0.5, 1.0, 2.5, and 5.0 µM LRRK2-IN-1 for 24 h. LRRK2-IN-1 suppressed MMP3, MMP13, iNOS, and COX2 and induced COL2 and SOX9 in a dose-dependent manner. **D** Quantitative analyses of the western blot results. Data represent mean ± SD; N = 3/group; *P < 0.05; **P < 0.01 by one-way ANOVA. **E** Immunofluorescence of iNOS, MMP13, and aggrecan expression in the IL-1β-induced chondrocytes treated with 5.0 µM LRRK2-IN-1 for 24 h. Scar bar: 400 μm

We investigated the cytotoxic effect of LRRK2-IN-1 on chondrocytes after 24-h treatment (Fig. 1A). LRRK2-IN-1 (0.5, 1.0, 2.5, and 5.0 µM) showed no appreciable inhibition on cell viability (Fig. 1B). To determine the anti-inflammatory and anti-catabolic effects of LRRK2-IN-1, chondrocytes that reached 80% confluency were treated with various concentrations of LRRK2-IN-1 with or without IL-1β (5 ng/mL) for 24 h and the cell lysate was subjected to western blot analyses. As shown in Fig. 1 C and 1D, a remarkable elevation in the expression levels of the inflammatory factors (e.g., COX2 and iNOS) and catabolic indicators (e.g. MMP3 and MMP13) in chondrocytes was induced by IL-1β, which could be dismissed by LRRK2-IN-1 in a dose-dependent way. Moreover, type II collagen (COL2, the main collagen component in cartilage) and SOX9 (the crucial regulator of chondrocyte phenotype and ECM cartilage homeostasis) were used as the anabolism indicators of chondrocytes. Western blot results revealed that COL2 and SOX9 expression were notably suppressed by IL-1β whereas LRRK2-IN-1 attenuated the suppression of IL-1β dose-dependently (Fig. 1 C and 1D). In addition, immunofluorescence analysis of the expression levels of iNOS, MMP13, and aggrecan revealed similar gene expression changes after the stimulation of IL-1β chondrocytes with or without LRRK2-IN-1 (5.0 µM) (Fig. 1E).

### Characterization of W-Exo-L@GelMA

**Fig. 2:**
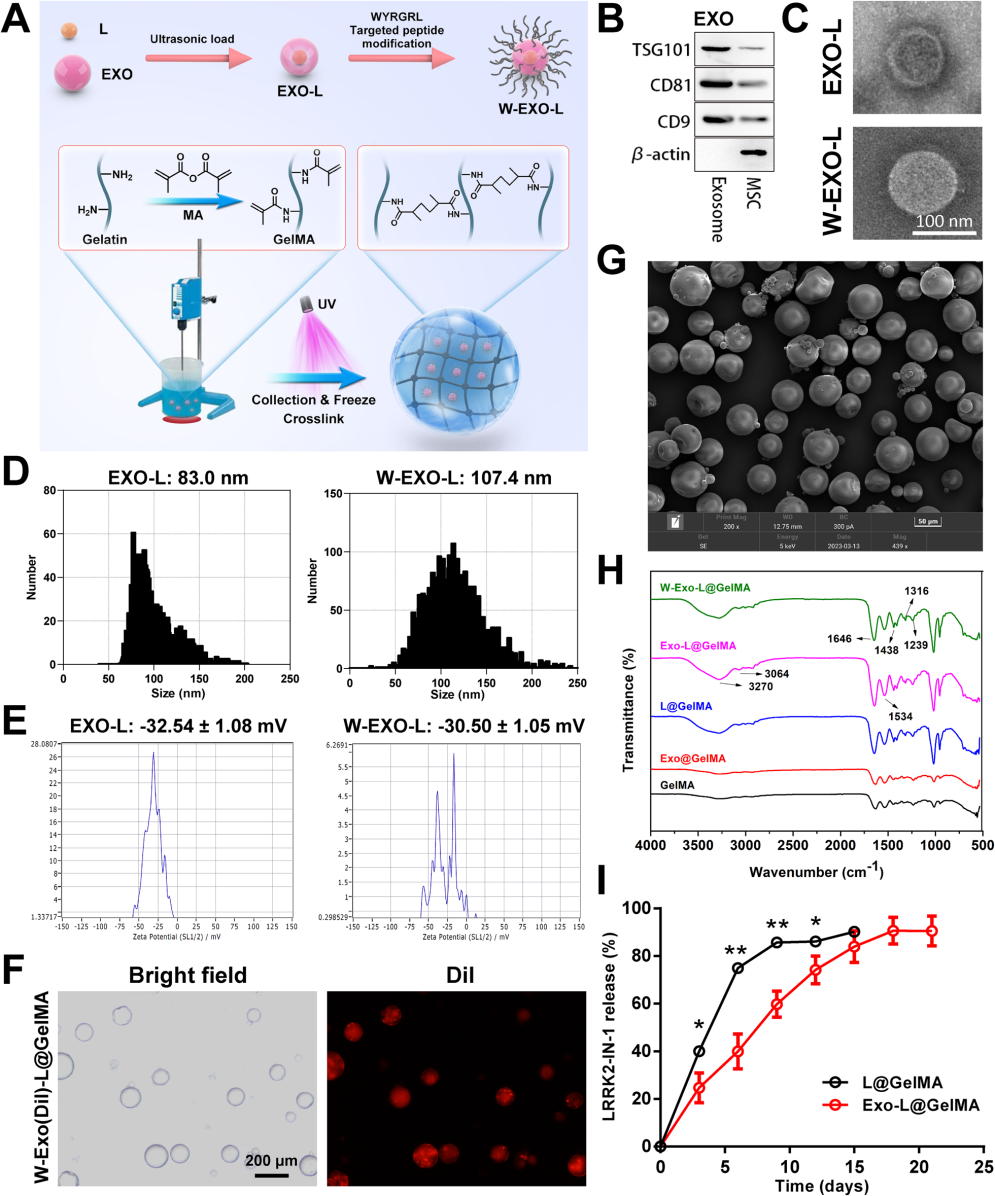
Characterization of W-Exo-L@GelMA. **A** The protocol of W-Exo-L@GelMA preparation. L, LRRK2-IN-1; EXO, exosome; W, cartilage affinity WYRGRL (W) peptide; UV, ultraviolet. **B** Western blot analyses showing that MSC exosomes could express characteristic marker proteins, such as TSG101, CD81, and CD9. **C** Scanning electron microscopy showing that the exosomes were round-shaped and the diameter of target peptide-modified exosomes increased compared with that of unmodified exosomes. **D** Diameter measurement of MSC exosomes. The diameter measurement showed the diameter of the exosomes after the modification of the targeted peptide increased compared with that of the unmodified exosomes. **E** Surface potential analysis of MSC exosomes. The surface potential of exosomes was increased after peptide modification. **F** Optical and fluorescent images of W-Exo-L@Gel. The exosomes containing Dil fluorescence were successfully encapsulated by GelMA microspheres with a size of about 100–200 μm. **G** Scanning electron microscopy showing the morphology and size of GelMA microspheres. **H** Infrared spectrum analysis of W-Exo-L@GelMA. The amide I (1646 cm^–1^), amide II (1534 cm^–1^), amide III (1316 cm^–1^), and amide A (3270 cm^–1^) and amide B (3064 cm^–1^), are peaks of infrared characterization of amide. 1285 cm^−1^ and 1420 cm^−1^ belong to C-N. **I** The drug release curve of Exo-L@GelMA in vitro. The drug release time of Exo-L@GelMA was prolonged by 1 week. Data represent mean ± SD; N = 3/group; *P < 0.05; **P < 0.01 by Student’s t-test

W-Exo-L@GelMA was prepared following the protocols shown in Fig. 2A. To confirm the intrinsic properties of exosomes, we checked the expression levels of the characteristic marker proteins of the mesenchymal stromal cell (MSC) exosomes we isolated. Western blot results showed that the MSC exosomes expressed characteristic marker proteins TSG101, CD81, and CD9, whereas they did not express β-actin (Fig. 2B). The morphology of the exosomes was further examined by electron microscopy (Fig. 2C). The exosomes were round-shaped and the targeted peptide-modified exosomes appeared larger than the unmodified exosomes. The diameter measurement showed the diameter of the exosomes after the modification of the targeted peptide increased (~ 107.4 nm) compared with that of unmodified exosomes (~ 83 nm) (Fig. 2D). The surface potential analysis showed that the surface potential of exosomes was increased after peptide modification (Fig. 2E). The encapsulation efficiency and loading capacity of LRRK2-IN-1 in exosomes are shown in Additional file 1: Tables S1, S2. The exosomes containing Dil fluorescence were successfully encapsulated by GelMA microspheres, as shown in Fig. 2F, with a size of about 100–200 μm. SEM showed the morphology and size of GelMA microspheres (Fig. 2G). As shown in Fig. 2H, the absorption spectrum bands of amide infrared radiation (IR), amide I (1646 cm^–1^), amide II (1534 cm^–1^), amide III (1316 cm^–1^), and amide A (3270 cm^–1^) and amide B (3064 cm^–1^), were peaks of infrared characterization of amide. 1285 cm^−1^ and 1420 cm^−1^ belong to the stretching vibration of C–N and the deformed vibration absorption peak of C-H of drugs respectively. The encapsulation efficiency and loading capacity of exosomes into GelMA is shown in Additional file 1: Tables S3, S4. The in vitro drug release curve showed that the drug release cycle was successfully prolonged by microsphere-encapsulated exosomes (Fig. 2I) and the release time was prolonged by 1 week compared with the microsphere without encapsulating exosomes.

### W-Exo-L@GelMA exhibits a strong chondrocyte-targeting effect and a pronounced action on promoting anabolism and suppressing catabolism and inflammation

**Fig. 3:**
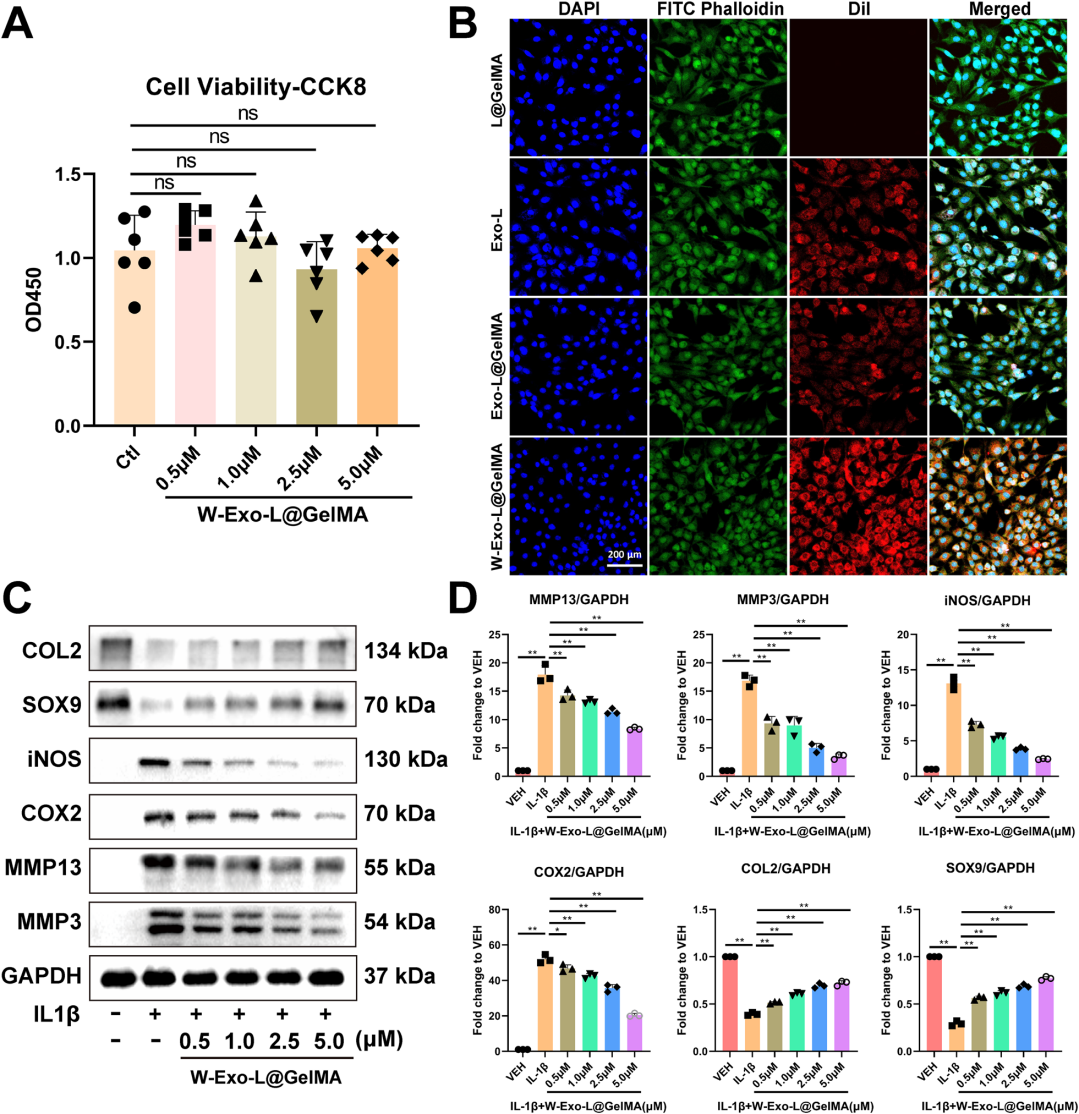
W-Exo-L@GelMA exhibits a strong chondrocyte-targeting effect and a pronounced action on promoting anabolism and suppressing catabolism and inflammation without causing the inhibition of chondrocyte viability. **A** Cell viability assessed by CCK8 assay. No obvious cytotoxicity on chondrocytes was observed when treated with W-Exo-L@GelMA loaded with 0.5, 1.0, 2.5, and 5.0 µM LRRK2-IN-1 for 48 h. Data represent mean ± SD; N = 6/group; one-way ANOVA; ns, not significant. **B** Immunofluorescence of Dil-labeled exosomes. The uptake of exosomes was observed in the chondrocytes when treated with Exo-L, Exo-L@GelMA or W-Exo-L@GelMA for 48 h. Dil was used for labeling exosomes (red), DAPI to label nuclei (blue), and Phalloidin to label the cytoskeleton (green). Scar bar: 200 μm. **C** Western blot analyses of the protein levels of anabolic, catabolic, and inflammatory factors in the IL-1β-induced chondrocytes treated with W-Exo-L@GelMA loaded with 0.5, 1.0, 2.5, and 5.0 µM LRRK2-IN-1 for 48 h. W-Exo-L@GelMA promoted COL2 and SOX9 and inhibited iNOS, COX2, MMP3, and MMP13 protein levels in a dose-dependent manner. **D** Quantitative analysis of the western blot results. Data represent mean ± SD; N = 3/group; *P < 0.05; **P < 0.01 by one-way ANOVA

The CCK8 assay was used to evaluate the in vitro cytotoxicity of W-Exo-L@GelMA on chondrocytes. As shown in Fig. 3A, no cytotoxicity was observed in the CCK8 assay, illustrating that the vast majority of the chondrocytes remained in a favorable condition throughout the 48-hour culture period which is important for the subsequent studies. Moreover, the cellular uptake activity of exosomes was tracked and localized using Dil fluorescence after 48-h incubation with L@GelMA, Exo-L, Exo-L@GelMA, or W-Exo-L@GelMA (Fig. 3B). The exosomes were distributed throughout the cytoplasm after being released from the hydrogel matrix (Fig. 3B). A significantly greater red fluorescence was observed in the W-Exo-L@GelMA group than in the Exo-L and Exo-L@GelMA groups, suggesting that the polypeptide WYRGRL successfully enhanced the cellular uptake of exosomes. In addition, as shown by western blot analyses, the IL-1β-induced upregulation of catabolic and inflammatory factors and downregulation of anabolic factors in chondrocytes were reversed in the LRRK2-IN-1-dependent manner after the treatment of W-Exo-L@GelMA loaded with 0.5, 1.0, 2.5, and 5.0 µM LRRK2-IN-1 for 48 h (Fig. 3 C and D).

### W-Exo-L@GelMA suppresses the IL-1β-induced transcriptomic responses related to catabolic effect, inflammation, and immune response

**Fig. 4:**
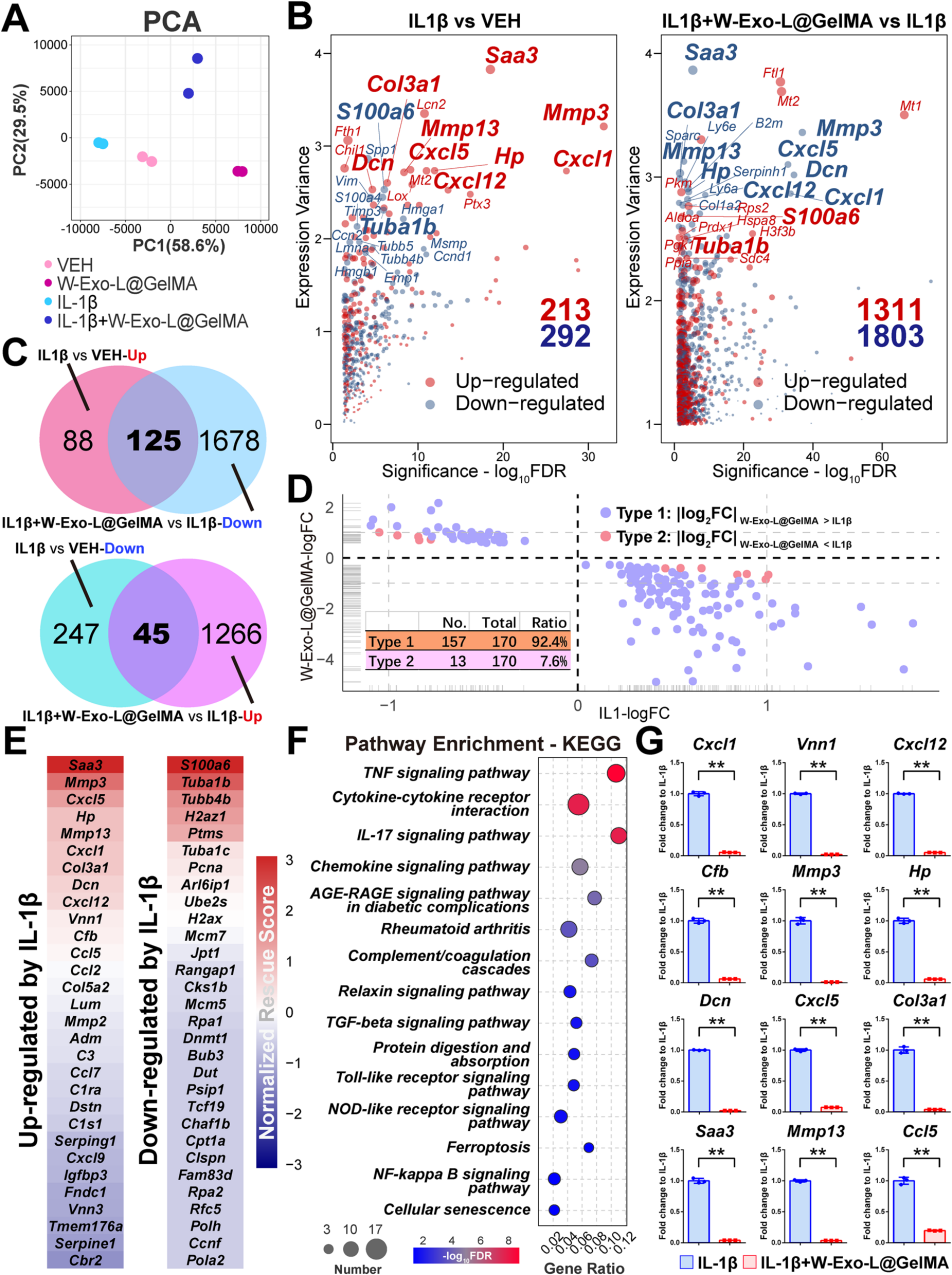
W-Exo-L@GelMA suppresses the IL-1β-induced transcriptomic responses related to catabolic effect, inflammation, and immune response. **A** Principal component analysis of the overall transcriptome of each sample. **B** Scatter plots of the differentially expressed genes (DEGs) in the comparison between the IL-1β and VEH groups or between the W-Exo-L@GelMA/IL-1β and IL-1β groups. The up- or down-regulated genes are indicated in red or blue color. The top 20 DEGs in the ranked gene list sorted in descending order of expression variance values are labeled by official gene symbols and the rescued genes are labeled in bold. Expression variance is defined by the absolute value of the difference in normalized gene expression (FPKM). **C** Overlap of the DEGs in each comparison. **D** The scatter plot of the completely-rescued (Type-1) and partially-rescued (Type-2) genes. **E** Top 30 IL-1β-induced or suppressed genes notably rescued by W-Exo-L@GelMA. **F** KEGG pathway analysis of the rescued genes (up-regulated by IL-1β and down-regulated by W-Exo-L@GelMA). **G** qPCR validations of the key rescued genes. Data represent mean ± SD; N = 3/group. **P < 0.01 by Student’s t-test

The PCA result showed the overall similarity and differences in the transcriptome of each sample (Fig. 4A). W-Exo-L@GelMA induced a transcriptomic shift in the PC1 axis, which was opposed to the effect of IL-1β on the transcriptome. Compared with the VEH group, 505 DEGs were identified in the IL-1β group, including 213 up-regulated and 292 down-regulated genes (Fig. 4B). In the comparison between the W-Exo-L@GelMA/IL-1β and IL-1β groups, we identified 3,114 DEGs, including 1,311 up-regulated and 1,803 down-regulated genes (Fig. 4B). Among the top 30 significant modified genes of IL-1β, *Saa3*, *Mmp3*, *Cxcl5*, *Hp*, *Cxcl1*, *Mmp13*, *Col3a1*, *Dcn*, and *Cxcl12* were notably up-regulated by IL-1β whereas they were all suppressed by W-Exo-L@GelMA (Fig. 4B). Moreover, the decreased expression of *S100a6* and *Tuba1b* induced by IL-1β was rescued by W-Exo-L@GelMA (Fig. 4B). Indeed, 58.7% of up-regulated genes (n = 125) and 15.4% of down-regulated genes (n = 45) were rescued by W-Exo-L@GelMA (Fig. 4C). Moreover, two types of rescued genes were further characterized by comparing the perturbation amplitudes of gene expression (fold-change values) (Fig. 4D). The Type-1 genes (n = 157; 92.4%) were defined as completely-rescued genes since the absolute value of fold changes in the comparison between the W-Exo-L@GelMA/IL-1β and IL-1β groups were larger than the ones in the comparison between the IL-1β and VEH groups. The Type-2 genes (n = 13; 7.6%) were defined as partially-rescued genes, which were much less than the Type-1 genes (Fig. 4D). To further characterize the most significant rescued genes and quantitate the rescued effect of W-Exo-L@GelMA on each gene, we used a robust rank aggregation (RRA) method to define a normalized rescued score. This score was used to identify the common polarized gene in the comparisons between the IL-1β and VEH groups or between the W-Exo-L@GelMA/IL-1β and IL-1β groups. According to the normalized rescued scores, the top 30 IL-1β-induced or suppressed genes were shown in Fig. 4E, which were notably rescued by W-Exo-L@GelMA. Besides catabolic genes such as *Mmp3*, *Mmp13*, and *Mmp2*, inflammation-related genes, such as C-X-C motif and C-C motif chemokine ligands, were identified in the top rescued gene list. To understand the pathways related to these recused genes (up-regulated by IL-1β and down-regulated by W-Exo-L@GelMA), we performed KEGG pathway enrichments. As shown in Fig. 4F, inflammation-related pathways, such as the TNF signaling pathway, Cytokine-cytokine receptor interaction, IL-17 signaling pathway, Chemokine signaling pathway, and NF-kappa B signaling pathway, were identified as significantly relevant to the rescued genes. Some OA-related pathways, such as the TGF-beta signaling pathway, Ferroptosis, and Cellular senescence, were also identified (Fig. 4F). These results suggested that W-Exo-L@GelMA suppressed the gene expression relate to IL-1β-induced catabolism, inflammation, and immune response. We further validated some key rescued genes, including *Mmp3*, *Mmp13*, *Cxcl1*, *Cxcl5*, *Cxcl12*, *Ccl5*, *Vnn1*, *Cfb*, *Hp*, *Dcn*, *Col3a1*, and *Saa3*, by qPCR (Fig. 4G).

### W-Exo-L@GelMA exhibits prolonged joint retention and attenuates the cartilage lesions and subchondral bone loss of the OA murine model

**Fig. 5:**
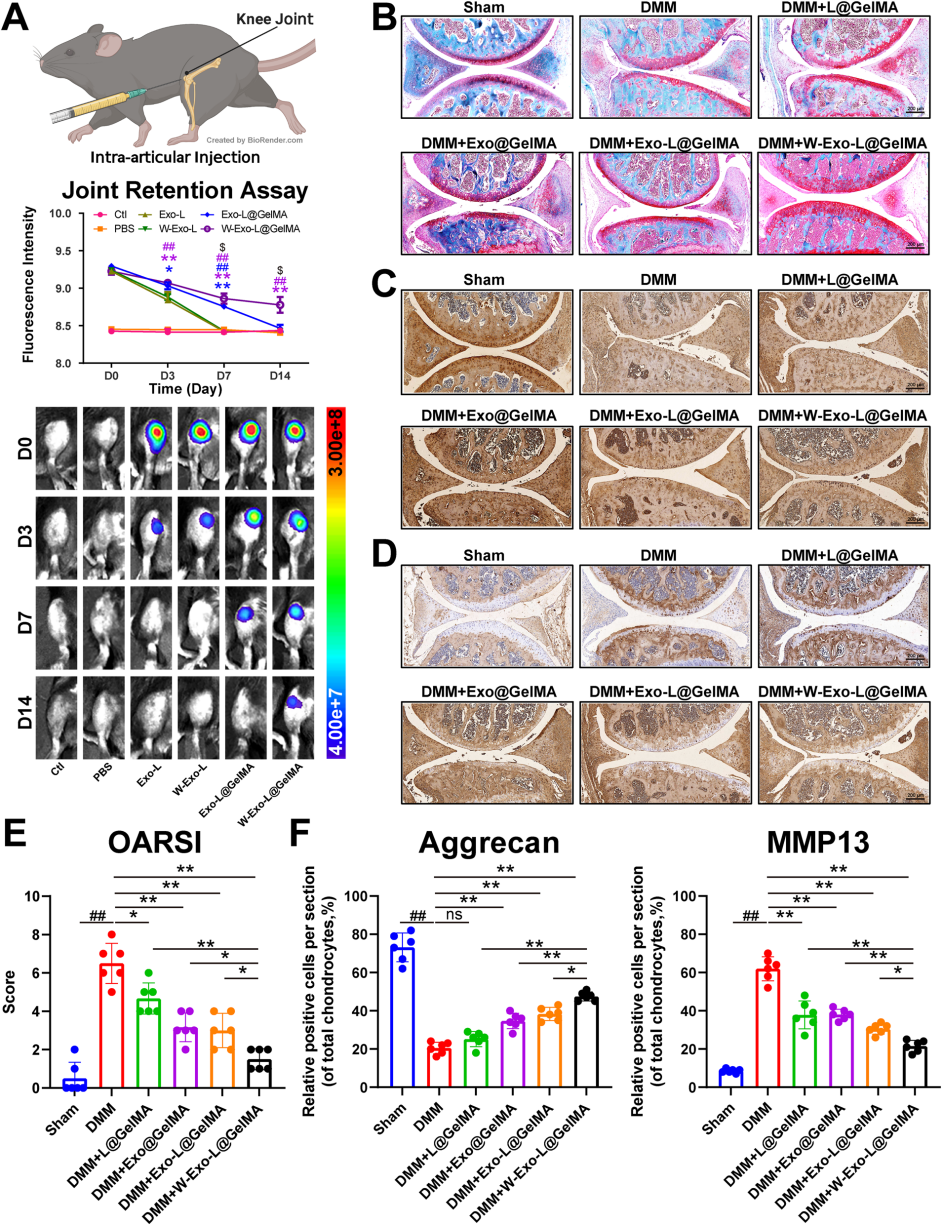
W-Exo-L@GelMA shows a significant joint retention effect and attenuates cartilage lesions in the OA murine model. **A** Joint retention assessed by in vivo fluorescence method. The hydrogel with Cy5-loaded exosomes or the Cy5-loaded exosomes alone was injected into the knee joint of the 12-week-old C57BL/6 mice. W-Exo-L@GelMA delayed the dissipation of the Cy5 fluorescence signal, indicating that the hydrogel can be retained 14 days after injection. Data represent mean ± SD; N = 3/group. **P < 0.01 by Student’s t-test; the colored groups versus Exo-L@GelMA. #P < 0.05; ##P < 0.01 by Student’s t-test, the colored groups versus W-Exo-L@GelMA; $P < 0.05 by Student’s t-test, W-Exo-L@GelMA versus Exo-L@GelMA. **B** Safranin O-fast green staining of knee sections. W-Exo-L@GelMA exhibited a stronger ability to attenuate cartilage wear and degeneration induced by destabilization of the medial meniscus (DMM) than L@GelMA, Exo@GelMA, or Exo-L@GelMA. Scar bar: 200 μm. **C** Immunohistochemistry representative images of aggrecan protein levels. Scar bar: 200 μm. Aggrecan expression was increased in local chondrocytes after the intervention, especially W-Exo-L@GelMA. **D** Immunohistochemistry representative images of MMP13 protein levels. Scar bar: 200 μm. The expression of MMP13 in local chondrocytes decreased after treatments. **E** Statistical analysis of OARSI histological scores. Data represent mean ± SD; N = 6/group. *P < 0.05; **P < 0.01 by one-way ANOVA. ##P < 0.01 by one-way ANOVA, the DMM group versus the control group. **F** Quantitative statistics of immunohistochemistry analyses of aggrecan and MMP13. Data represent mean ± SD; N = 6/group. *P < 0.05; **P < 0.01 by one-way ANOVA. ##P < 0.01 by one-way ANOVA, the DMM group versus the control group

Based on the notable results in vitro, the W-Exo-L@GelMA was further evaluated in vivo when applied to the treatment of OA. To investigate whether W-Exo-L@GelMA enhances the retention ability of exosomes in vivo, we used Cy5 to label the exosomes and detected the Cy5 fluorescence by an in-vivo imaging system (IVIS). On the seventh day after injections, the Cy5 fluorescence disappeared in the W-Exo-L and Exo-L groups, whereas it could be detected in the Exo-L@GelMA and W-Exo-L@GelMA groups **(**Fig. 5A**)**, indicating that the GelMA hydrogel prolonged the exosome retention in the joint cavity. On the fourteenth day, the fluorescence was only detected in the W-Exo-L@GelMA group **(**Fig. 5A**)**, indicating that the targeting peptide modification further prolonged the retention of exosomes. These findings suggested that W-Exo-L@GelMA had a favorable ability to preserve exosomes and target cartilage in vivo, which is consistent with the in vitro findings.

To assess the therapeutic effects in vivo, we employed the DMM-induced murine OA model. The DMM mice were randomly assigned into five groups to receive intra-articular injections of 10 µL saline, L@GelMA, Exo@GelMA, Exo-L@GelMA, or W-Exo-L@GelMA every 2 weeks for 2 months. As for the Sham group, the mice received 10 µL saline solution as control. Safranin O-fast green staining illustrated that severe articular cartilage degeneration, characterized by defects and fissures, disorganized chondrocyte sequence, and ECM loss, was found in the DMM group, which was consistent with the elevated OARSI score of the DMM group (Fig. 5B and E). These findings indicated a significant progression of OA induced by DMM. The hybrid hydrogel groups showed attenuated cartilage lesions and decreased OARSI scores compared with the DMM group (Fig. 5B and E). Of note, the W-Exo-L@GelMA group demonstrated the most efficient therapeutic effects against DMM-induced cartilage degeneration than the L@GelMA, Exo@GelMA, or Exo-L@GelMA, suggesting that the targeted function of WYRGRL peptide facilitated an enhanced therapeutic effect of W-Exo-L@GelMA. Moreover, immunohistochemistry staining showed that the aggrecan expression was elevated and the MMP13 expression was decreased in the treated groups compared with the DMM group. The W-Exo-L@GelMA group showed the stronger effect on increasing aggrecan and suppressing MMP13 than the Exo-L@GelMA group (Fig. 5 C, D, and F).

**Fig. 6:**
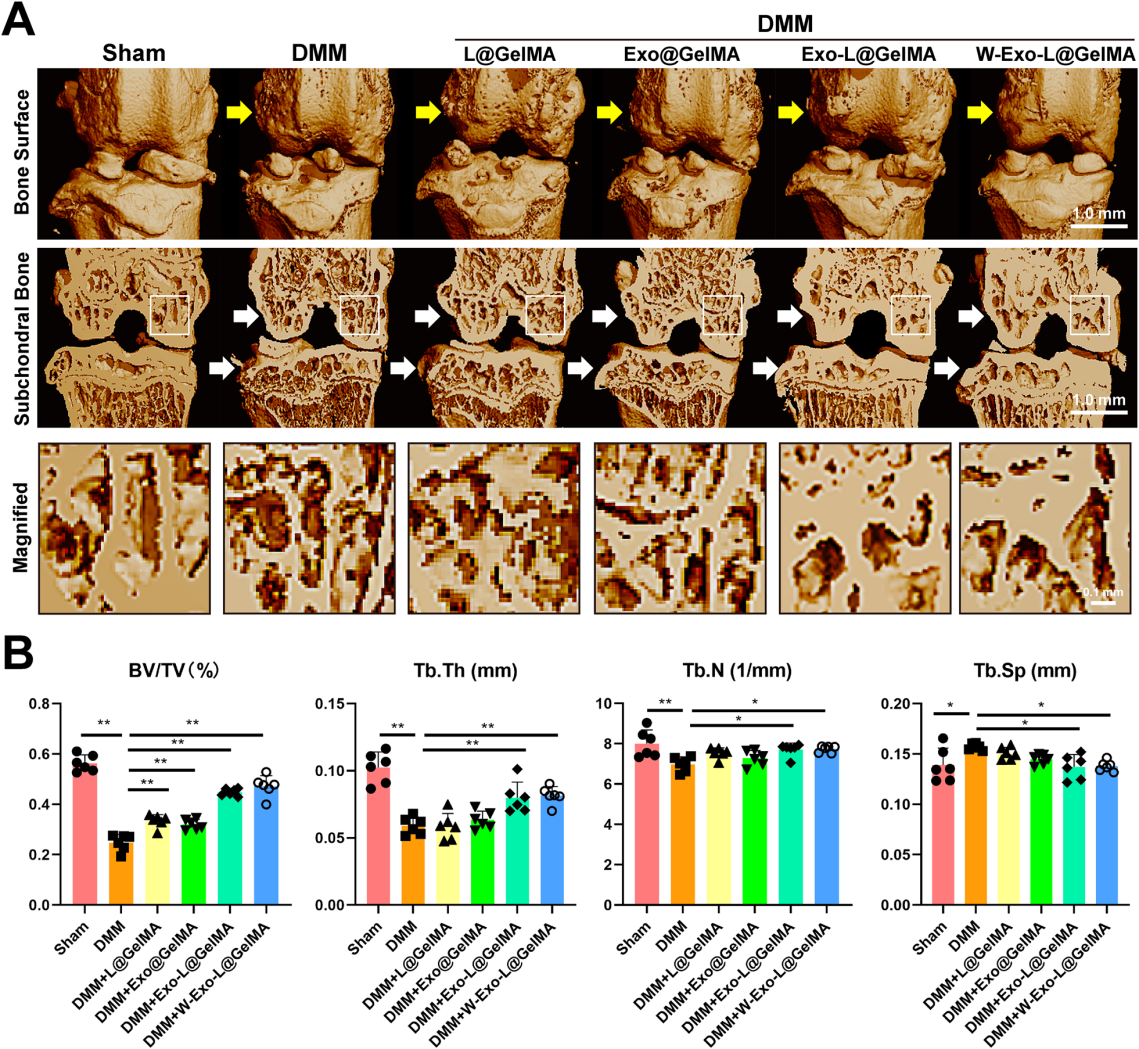
W-Exo-L@GelMA inhibits osteophyte formation and subchondral bone loss in the OA murine model. **A** Micro-CT representative images of the bone surface and the coronal plane of the subchondral bone of joints. W-Exo-L@GelMA notably reduced the osteophyte formation of the surface and the loss of subchondral bone. **B** Quantitative analysis of the parameters of tibial subchondral bone: trabecular number (Tb. N), volume/tissue volume (BV/TV), trabecular thickness (Tb. Th), and trabecular separation (Tb. Sp). Data represent mean ± SD; N = 6/group. *P < 0.05; **P < 0.01 by one-way ANOVA

Since subchondral bone is a critical element of the cartilage-subchondral bone unit, we assessed the changes in subchondral bone by micro-CT analysis. The 3D images of the joints showed that the obvious osteophyte formation existed on the bone surface of the DMM group whereas the osteophyte formation was reduced in the W-Exo-L@GelMA group (Fig. 6A). Moreover, the coronal section of the subchondral bone showed that DMM-induced typical subchondral bone loss of the femur and tibia related to OA whereas Exo-L@GelMA and W-Exo-L@GelMA notably preserved the subchondral bone of the femur and tibia (Fig. 6A). The quantitative analyses showed that Exo-L@GelMA and W-Exo-L@GelMA exhibited a more efficient action in increasing Tb. N and Tb. Th and decreasing Tb. Sp than the other groups, thus leading to an increased BT/TV compared with the DMM and the other treatment groups (Fig. 6B). Although W-Exo-L@GelMA showed no significantly stronger effects on subchondral bone than Exo-L@GelMA, W-Exo-L@GelMA had a tendency of increasing bone mass.

### W-Exo-L@GelMA promotes aggrecan and suppresses MMP13 expression in cultured human OA cartilage explants

**Fig. 7:**
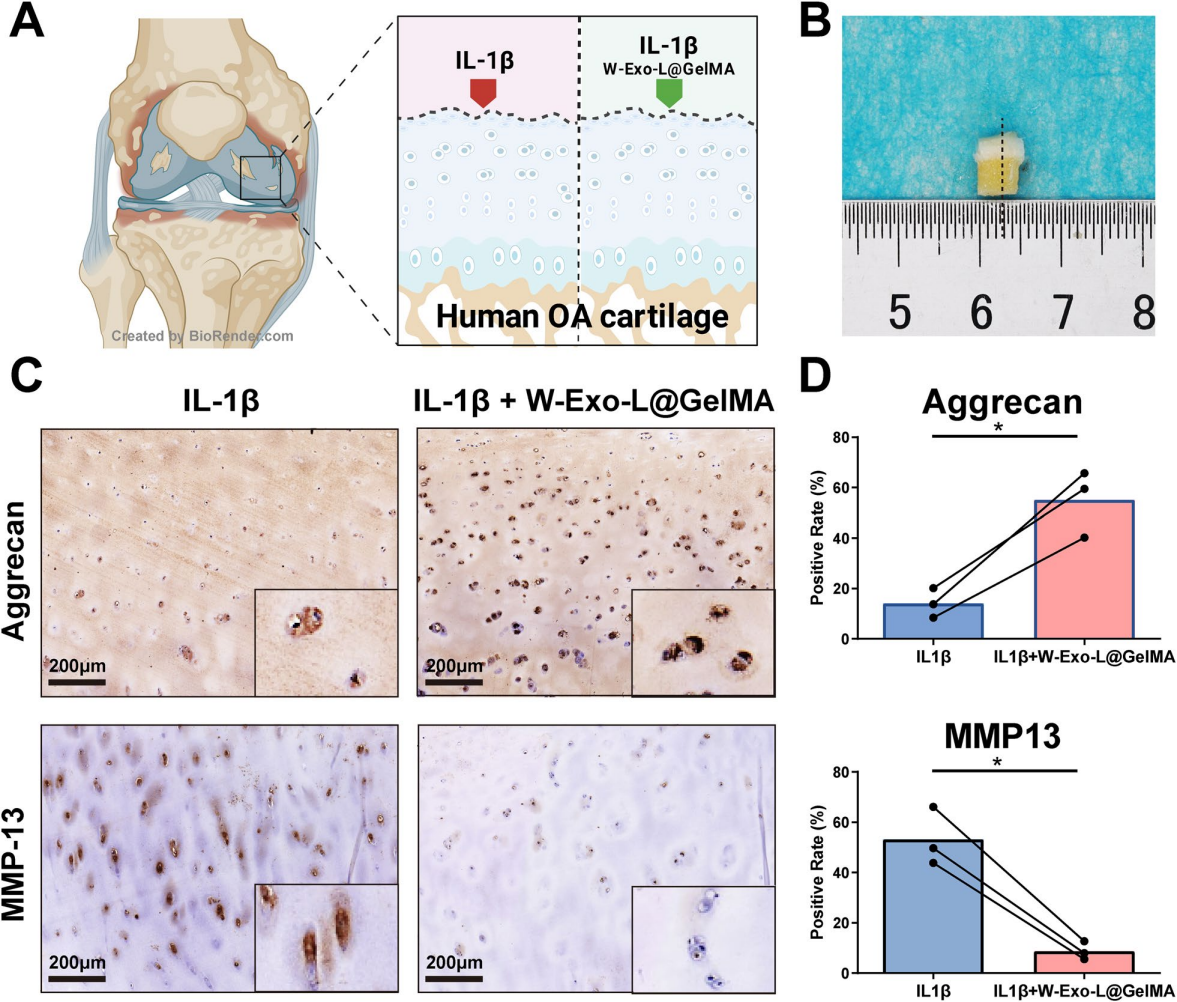
W-Exo-L@GelMA exhibits a therapeutic effect on human OA cartilage explants by promoting aggrecan and suppressing MMP13 expression. **A** Schematic diagram of the procedures of the human OA cartilage explant harvest and treatments. The human OA cartilage explants were harvested from the medial condyle of the femur of the OA patients undergoing total knee arthroplasty. **B** Representative image of the harvested human OA cartilage explant. **C** Representative images of Immunohistochemistry analyses of the aggrecan and MMP13 expression levels in the IL-1β- maintained explants treated with or without W-Exo-L@GelMA (5.0 µM) for 72 h. Scar bar: 200 μm. **D** Quantitative analyses of immunohistochemistry. Data represent mean ± SD; N = 3/group. *P < 0.05 by paired t-test

To investigate the potential of W-Exo-L@GelMA in clinical applications, we assess its effect on the aggrecan and MMP13 expression in the cultured human OA cartilage explants harvested from the medial femoral condyle of TKA patients (Fig. 7A). As shown in Fig. 7B, the size of the cartilage explants was around 5 × 5 × 5 mm^3^ and the cartilage thickness was around 2 mm. Then, the cartilage explants from the same patients were divided into two parts, which were treated with or without W-Exo-L@GelMA loaded with 5.0 µM LRRK2-IN-1 for 72 h, respectively (Fig. 7A). To maintain the OA phenotypes of gene expression, the cartilage explants were cultured in the medium containing human IL-1β (10 ng/mL). The immunohistochemistry analyses showed that the 72-hour treatment of W-Exo-L@GelMA was efficient to increase the aggrecan expression and suppress the MMP13 expression in the human OA cartilage, indicating the potential of W-Exo-L@GelMA in human OA treatment (Fig. 7B and D).

## Discussion

Pharmacotherapy targeting OA cartilage is one of the core missions in OA management [32, 46]. However, the joint is a challenging arena for drug delivery. One of the obstacles hindering the achievement of pronounced therapeutic effects is the inability to deliver drugs efficiently to OA cartilage by systemic administration. Thus, intra-articular injections directly into the joints of the OA are preferred, whereas the rapid cellular and enzymatic clearance of drugs after intra-articular injection cause low accumulation and retention of drugs in the joint cavity. The leakage from the joint or excretion from synovial blood vessels and lymphatic vessels are also the reasons for the short half-life and residence time in the joint [47, 48]. Moreover, the dense packing of collagen and proteoglycans in joint tissue as well as the highly negatively charged glycosaminoglycans form a critical barrier needed to be penetrated in cartilage, which increases the difficulty in drug delivery [49, 50]. The complexity of these factors makes chondrocyte-targeted drug delivery challenging. Up to now, various cartilage-targeting nanoplatforms have been developed which are beneficial in overcoming the dense collagen barrier and facilitating drug delivery [49, 50]. Of note, the nanoparticles with a chondrocyte-affinity peptide can remain within cartilage for a prolonged period, making this an ideal substance for treating osteoarthritis.

Exosomes are natural nanovesicle that many cells secrete to facilitate communication between cells and to transmit substances such as proteins, nucleic acids, and even synthetic drugs [51, 52]. In this emerging field of drug delivery research, exosomes are modified on the surface to enhance their functionality for site-specific drug delivery as well as in vivo imaging and tracking [53]. Exosomes have the potential to be modified and connected to peptides or ligands through the genomic editions of exosome-derived cells, direct modification (such as click chemistry), and other methods, notably enhancing the engineerability of exosomes [18, 54, 55]. The genomic edition method is time-consuming and technically challenging. Before the collection of exosomes, the fusion plasmids containing the corresponding gene of the target peptide or ligand need to be prepared and the lentiviral vector constructed, followed by the infection of cell lines. In this context, click chemistry methods exhibit advantages since the peptides are connected directly to the exosome membrane by chemical reactions. In our study, methacrylic anhydride was amidated with amino groups on the surface of exosomes to form olefin double bond-modified exosomes which were then amidated with amino groups in targeting peptides to achieve the combination of polypeptides and exosomes. MSC-derived exosomes were used as drug-loaded nanocarriers in our design. Exosomes derived from MSCs have been increasingly used in the treatment of OA in recent years due to their properties of promoting cartilage repair and extracellular matrix synthesis [56–58]. An exosome surface-conjugated collagen II-targeting peptide (WYRGRL) was then used to develop drug-delivery nanoplatforms targeting cartilage [59, 60]. We designed WYRGRL-modified exosomes encapsulated with hydrogels to achieve LRRK2-IN-1 targeted delivery deep into the cartilage, and prolong drug retention by exploiting the superior tissue penetration capability of exosomes and type II collagen-targeting peptide. It is believed that targeted drug delivery provides many benefits, including improved therapeutic efficacy, reduced toxicity to other tissues, and a reduction in treatment costs. Moreover, encapsulating the medication within exosomes can avoid the phagocytosis of monocytes and the degradation of enzymes in the cell matrix in vivo.

Gelatin, as a product of natural collagen, is the main component of extracellular matrix in the body. Methacrylic acid modified gelatin (GelMA) has good biological properties and adjustable physical properties, and is widely used in tissue engineering in bone, cartilage, heart and other fields. GelMA is a highly attractive hydrogel with photocrosslinking properties, making it successful in maintaining suitable biological properties, tunable physical characteristics, and pleiotropic collaboration functions with other delivery systems [61, 62]. GelMA coating is a critical technical improvement of exosome-based drug delivery in this study. GelMA microsphere hydrogel could be successfully fabricated based on the photocrosslinking reaction. Traditional crosslinking agents, including glutaraldehyde and formaldehyde, are usually limited due to toxicity and low crosslinking efficiencies [63]. In contrast, LAP is a green crosslinker with good degradability, high reaction efficiencies, and no cytotoxicity, which are highly desirable for in vivo applications. FT-IR results indicate that GelMA microsphere hydrogel was successfully prepared without the formation of chemical bonds. GelMA microsphere hydrogel significantly reduced the drug release rate, which was largely ascribed to the covalent crosslinking of gelatin molecules (secondary amide bond) [64]. Moreover, the methacrylic acid modified gelatin water gel contains RGD peptide that stimulates cell adhesion, which can effectively establish an immune isolation barrier and significantly enhance the stability of transplanted exosomes [65, 66]. Therefore, GelMA is an effective way to maintain the biological activity of exosomes.

In this study, GelMA microsphere hydrogel successfully improved the joint retention of exosomes, thus enhancing the treatment effects in vivo. It is demonstrated that the structure of hydrogel plus targeting peptide can effectively retain fluorescent signals in exosomes for more than 14 days, overcoming the limitations of fast clearance and low retention rates of exosomes. Moreover, the targeting peptide provided the drug-loaded exosomes released from the GelMA microspheres with a moving direction, which facilitated their phagocytosis by chondrocytes. Due to the long-term, stable, and effective drug action, W-Exo-L@GelMA showed the best therapeutic effect for improving DMM-induced OA in mice.

As a critical achievement of this study, W-Exo-L@GelMA is characterized by many excellent properties, including a small diameter, good potential, and ideal cartilage affinity, enabling it to effectively overcome the dense type II collagen barrier and exert precise therapeutic effects on OA. We present here a simple, rapid, and biorthogonal chemical approach for delivering disease-modifying drugs by modifying the exosome surface with a cartilage-targeting peptide for targeting and retention, demonstrating that cartilage-targeting and exosome-mediated drug delivery may offer a novel strategy for cell-free OA treatment. Overall, our delivery hydrogel approach demonstrates targeted and drug-loaded exosome protection. In addition, due to a simple production process and edible synthetic ingredients, our delivery hydrogel has significant therapeutic potential in clinical settings.

## Conclusions

In this study, we designed a photocrosslinking spherical hydrogel-encapsulated targeting peptide-modified engineered exosomes and investigated the performance of this composite spherical hydrogel loaded with LRRK2-IN-1 in vitro and in vivo. We successfully enhanced the targeting ability and retention of exosomes using a series of strategies, including the surface modification of cartilage affinity peptide and the encapsulating of photocrosslinking spherical hydrogel, which notably overcome the limitation of fast clearance and low retention of exosomes. W-Exo-L@GelMA exhibited considerable potential in OA attenuation and cartilage repair which may be an advanced nanotechnology-based strategy for OA treatment.

### Supplementary Information

**Additional file 1: Fig. S1.** The detailed composition of targeting peptide sequence. The targeting peptide sequence is WYRGRL with 849.98 molecular weight. HPLC analysis showed the peptide purity is 99.07%. The specific parameters: HPLC Column Kromasil 100-5C18 (4.6 mm * 150 mm, 5 micron); Detection wavelength: 220 nm; Gradient 10–70% A in 25 min; Mobile phase buffer A: 0.1% TFA + 100% CH3CN; Buffer B: 0.1% TFA + 100% H2O. **Table S1.** Determination of LRRK2-IN-1 loading capacity of Exo. **Table S2.** Determination of encapsulation efficiency of LRRK2-IN-1 in Exo. **Table S3.** Determination of encapsulation efficiency of exosomes into GelMA. **Table S4.** Determination of loading capacity of exosomes into GelMA.

## Data Availability

The data are available from the corresponding author upon reasonable request.
