# Supplementary material for: Injectable photocrosslinking spherical hydrogel-encapsulated targeting peptide-modified engineered exosomes for osteoarthritis therapy
Source: J Nanobiotechnology. 2023 Aug 21;21:284. doi: 10.1186/s12951-023-02050-7 (PMC10440922; doi:10.1186/s12951-023-02050-7)
Supplement: Supplementary file 1 — Additional file 1: Fig. S1. The detailed composition of targeting peptide sequence. The targeting peptide sequence is WYRGRL with 849.98 molecular weight. HPLC analysis showed the peptide purity is 99.07%. The specific parameters: HPLC Column Kromasil 100-5C18 (4.6 mm * 150 mm, 5 micron); Detection wavelength: 220 nm; Gradient 10–70% A in 25 min; Mobile phase buffer A: 0.1% TFA + 100% CH3CN; Buffer B: 0.1% TFA + 100% H2O. Table S1. Determination of LRRK2-IN-1 loading capacity of Exo. Table S2. Determination of encapsulation efficiency of LRRK2-IN-1 in Exo. Table S3. Determination of encapsulation efficiency of exosomes into GelMA. Table S4. Determination of loading capacity of exosomes into GelMA. [file 12951_2023_2050_MOESM1_ESM.docx]

**Injectable Photocrosslinking Spherical Hydrogel-Encapsulated Targeting Peptide-Modified Engineered Exosomes for Osteoarthritis Therapy**

Junlai Wan ^a†^, Zhiyi He ^a†^, Renpeng Peng ^a†^, Xiaopei Wu ^b^, Ziqing Zhu ^a^, Jiarui Cui ^c^, Xiaoxia Hao ^d^, Anmin Chen ^a*^, Jiaming Zhang ^a*^, Peng Cheng ^a*^

^a^ Department of Orthopedics, Tongji Hospital, Tongji Medical College, Huazhong University of Science and Technology, Wuhan 430030, China

^b^ State Key Laboratory of Advanced Technology for Materials Synthesis and Processing, Wuhan University of Technology, Wuhan 430070, China

^c^ Longhua Hospital, Shanghai University of Traditional Chinese Medicine, Shanghai 200032, China.

^d^ Department of Rehabilitation, Tongji Hospital, Tongji Medical College, Huazhong University of Science and Technology, Wuhan 430030, China

^†^ These authors contributed equally to this work.

^*^ Correspondence should be addressed to Prof. Anmin Chen, Dr. Peng Cheng, and Dr. Jiaming Zhang.

E-mail: [chengpeng2015@tjh.tjmu.edu.cn](mailto:chengpeng2015@tjh.tjmu.edu.cn) (P. Cheng); [anminchen@hust.edu.cn](mailto:anminchen@hust.edu.cn) (A. Chen); [jiaming_zhangtjmc@icloud.com](mailto:jiaming_zhangtjmc@icloud.com) (J. Zhang: 0000-0001-8520-566X)

Fax: +86-027-83662640 Tel: +1-8868259760

Postal address: 1095#, Jie-Fang Avenue, Qiaokou District, Wuhan, Hubei 430030, China


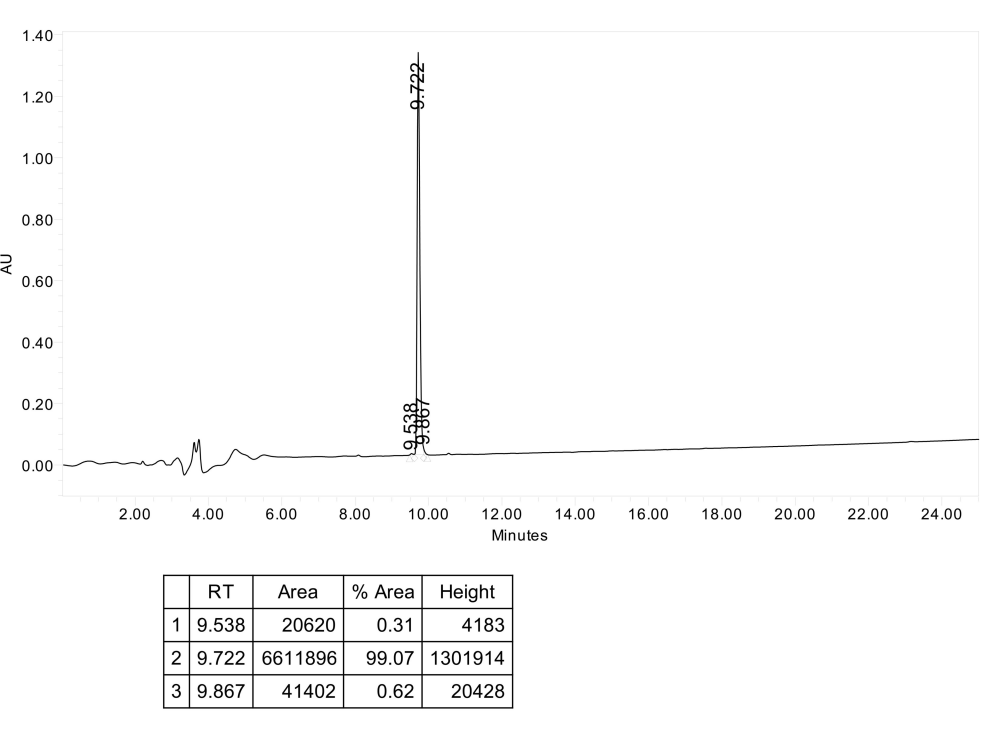


**Figure S1. The detailed composition of targeting peptide sequence.** The targeting peptide sequence is WYRGRL with 849.98 molecular weight. HPLC analysis showed the peptide purity is 99.07%. The specific parameters: HPLC Column Kromasil 100-5C18 (4.6 mm * 150 mm, 5 micron); Detection wavelength: 220 nm; Gradient 10-70% A in 25 min; Mobile phase buffer A: 0.1% TFA + 100% CH_3_CN; Buffer B: 0.1%TFA + 100%H_2_O.

**Table S1. Determination of encapsulation efficiency of LRRK2-IN-1 in Exo**

| LRRK2-IN-1 (μg/mL) | Exo  (μg/mL) (W_a_) | Concentration  (μg/mL) (W_b_) | Encapsulation Efficiency (%) | Mean ± SD  (%) |
| --- | --- | --- | --- | --- |
| 400.00 | 400.00 | 210.78 | 52.70 | 49.95 ± 2.58 |
| 400.00 | 400.00 | 198.26 | 49.57 |  |
| 400.00 | 400.00 | 190.32 | 47.58 |  |

Exo, exosome; SD, standard deviation. Drug encapsulation efficiency =W_b_/W_a_×100%. W_a_ is the mass of the drug loaded, and W_b_ is the initial system mass of the drug.

**Table S2. Determination of LRRK2-IN-1 loading capacity of Exo**

| Exo/LRRK2-IN-1  (μg/mL) (W_0_) | Concentration  (μg/mL) (W_1_) | Loading rate  (%) | Mean ± SD  (%) |
| --- | --- | --- | --- |
| 610.78 | 210.78 | 34.51 | 33.30 ± 1.14 |
| 598.26 | 198.26 | 33.14 |  |
| 590.32 | 190.32 | 32.24 |  |

Exo, exosome; SD, standard deviation. Drug loading rate=W_1_/W_0_×100%. W_1_ is the mass of the drug loaded, and W_0_ is the total mass of the drug-loaded exosomes.

**Table S3. Determination of** **encapsulation efficiency of exosomes into GelMA**

| Exo  (μg/mL) (W_a_) | GelMA  (g) | Protein Concentration (μg/mL) (W_b_) | Encapsulation Efficiency (%) | Mean ± SD  (%) |
| --- | --- | --- | --- | --- |
| 300 | 1.00 | 147.22 | 49.07 | 50.22 ± 1.08 |
| 300 | 1.00 | 151.14 | 50.38 |  |
| 300 | 1.00 | 153.65 | 51.22 |  |

GelMA, Gelatin-Methacryloyl; Exo, exosome; SD, standard deviation. Exosome encapsulation efficiency =W_b_/W_a_×100%. W_a_ is the mass of the exosome loaded, and W_b_ is the initial system mass of the exosome.

**Table S4. Determination of loading capacity of exosomes into GelMA**

| GelMA/Exo  (μg/mL) (W_0_) | Protein Concentration  (μg/mL) (W_1_) | Loading rate  (%) | Mean ± SD  (%) |
| --- | --- | --- | --- |
| 1147.22 | 147.22 | 12.83 | 13.09 ± 0.24 |
| 1151.14 | 151.14 | 13.12 |  |
| 1153.65 | 153.65 | 13.32 |  |

GelMA, Gelatin-Methacryloyl; Exo, exosome; SD, standard deviation. Exosome loading rate=W_1_/W_0_×100%. W_1_ is the mass of the exosome loaded, and W_0_ is the total mass of the exosome-loaded GelMA.
